# 2-Bromo-4-*tert*-butyl-6-[(pyridin-2-yl­imino)­meth­yl]phenol

**DOI:** 10.1107/S1600536811050148

**Published:** 2011-11-30

**Authors:** V. Balasubramani, T. Vinuchakkaravarthy, Sreeraj Gopi, S. Narasimhan, D. Velmurugan

**Affiliations:** a Asthagiri Herbal Research Foundation, 162-A, Industrial Estate, Perungudi, Chennai 600 092, India; b Centre of Advanced Study in Crystallography and Biophysics, University of Madras, Maraimalai Campus (Guindy Campus), Chennai 600 025, India

## Abstract

In the title compound, C_16_H_17_BrN_2_O, the pyridine and benzene rings are almost coplanar [dihedral angle = 1.3 (2)°]. An intra­molecular O—H⋯Br inter­action forms an *S*(5) ring motif.

## Related literature

For the anti-bacterial and anti-tumor activity of substituted salicyl­aldehyde derivatives, see: Jesmin *et al.* (2010 ▶); Pelttari *et al.* (2007 ▶) and for the biological activity of 2-amino­pryidine derivatives, see: Hagmann *et al.* (2000 ▶). For related structures, see: Puthilibai *et al.* (2008 ▶); Phurat *et al.* (2010 ▶); Wang *et al.*(2010 ▶). For the synthesis, see: Pannerselvam *et al.* (2005 ▶).
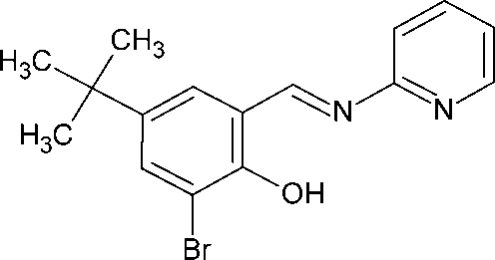


## Experimental

### 

#### Crystal data

C_16_H_17_BrN_2_O
*M* *_r_* = 333.23
Monoclinic, 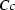

*a* = 10.0241 (11) Å
*b* = 16.1355 (16) Å
*c* = 9.4308 (13) Å
β = 92.050 (6)°
*V* = 1524.4 (3) Å^3^
*Z* = 4
Mo *K*α radiation
μ = 2.69 mm^−1^
*T* = 293 K
0.2 × 0.2 × 0.2 mm

#### Data collection

Bruker SMART APEXII area-detector diffractometer
6913 measured reflections
3051 independent reflections
2564 reflections with *I* > 2σ(*I*)
*R*_int_ = 0.035

#### Refinement

*R*[*F* ^2^ > 2σ(*F* ^2^)] = 0.033
*wR*(*F* ^2^) = 0.081
*S* = 0.98
3051 reflections
184 parameters
2 restraints
H-atom parameters constrained
Δρ_max_ = 0.50 e Å^−3^
Δρ_min_ = −0.37 e Å^−3^
Absolute structure: Flack (1983 ▶), 1147 Friedel pairs
Flack parameter: 0.009 (9)

### 

Data collection: *APEX2* (Bruker, 2008 ▶); cell refinement: *SAINT* (Bruker, 2008 ▶); data reduction: *SAINT*; program(s) used to solve structure: *SHELXS97* (Sheldrick, 2008 ▶); program(s) used to refine structure: *SHELXL97* (Sheldrick, 2008 ▶); molecular graphics: *ORTEP-3* (Farrugia, 1997 ▶); software used to prepare material for publication: *SHELXL97* and *PLATON* (Spek, 2009 ▶).

## Supplementary Material

Crystal structure: contains datablock(s) global, I. DOI: 10.1107/S1600536811050148/im2341sup1.cif

Structure factors: contains datablock(s) I. DOI: 10.1107/S1600536811050148/im2341Isup2.hkl

Supplementary material file. DOI: 10.1107/S1600536811050148/im2341Isup3.cml

Additional supplementary materials:  crystallographic information; 3D view; checkCIF report

## Figures and Tables

**Table 1:** Hydrogen-bond geometry (Å, °)

*D*—H⋯*A*	*D*—H	H⋯*A*	*D*⋯*A*	*D*—H⋯*A*
O1—H1⋯Br1	0.82	2.46	3.021 (3)	127

**Fig. 1.**
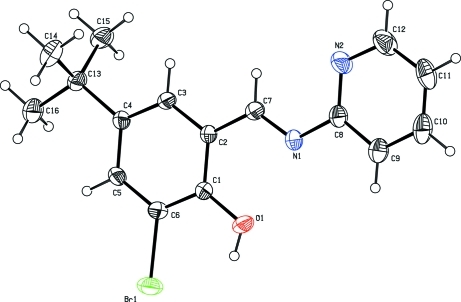
Molecular structure of the title compound, showing displacement ellipsoids drawn at the 30% probability level. H atoms are presented as a small spheres of arbitrary radius.

## supplementary crystallographic information

### Comment

The crystal structure determination of the title compound was undertaken as a part of the synthesis, structure and properties of new class of substituted salicylaldehyde derivatives.

In the crystal structure the pyridine ring and the substituted phenyl rings are essentially co-planar with a mean deviation of 0.0057Å and 0.0053Å, respectively, from the least square planes of the corresponding constituent ring atoms. Unlike the other structures, the N(1) atom of the pyridine ring aligns with the plane of the other atoms contributing the ring (C12—C13—C14—C15—C16). The dihedral angle between pyridine ring and the phenyl ring is 1.3 (2)°. The Br(1) atom is almost co-planar with the phenyl ring (C1 to C6) with a mean deviation of 0.025 (1)Å. An intramolecular O(1)—H···Br(1) hydrogen bond forms a S(5) ring motif. Intramolecular C(7)—H···N(2) weak interaction is also observed in the structure.

### Experimental

The synthesis of the title compound follows the modified method of Schiff's base prepartion described by Pannerselvam *et al.* (2005). The microwave-assisted condensation of 3-bromo-5-*tert*-btuyl-2-hydroxybenzaldehyde and 2-amino pyridine was carried out in a domestic oven, Samsung SMH9151BE. Equimolar concentrations of 3-bromo-5-*tert*-butyl-2-hydroxy benzaldehyde and 2-amino pyridine (3mmol each) were dissolved in anhydrous methanol (5mL) at ambient temperature in an 25mL Erlenmeyer flask. The mixture was subjected to microwave irradiation for an optimized time (8 mins) on the M-High setting (800W). It was then cooled and diluted with ice-cold water. The product yield was found to be 72% and the purity was checked using TLC. The compound was re-crystallized from methanol/water mixture at room temperature to yield single crystals.

### Refinement

Hydrogen atoms were placed in calculated positions with C—H = 0.93Å and refined using the riding model approximation with a fixed isotropic displacement parameter of *U*_iso_(H) = 1.6 *U*_eq_(C).

### Crystal data

**Table:**

C_16_H_17_BrN_2_O	*F*(000) = 680
*M**_r_* = 333.23	*D*_x_ = 1.452 Mg m^−^^3^
Monoclinic, *C**c*	Mo *K*α radiation, λ = 0.71073 Å
Hall symbol: C -2yc	Cell parameters from 3053 reflections
*a* = 10.0241 (11) Å	θ = 2.4–28.3°
*b* = 16.1355 (16) Å	µ = 2.69 mm^−^^1^
*c* = 9.4308 (13) Å	*T* = 293 K
β = 92.050 (6)°	Block, red
*V* = 1524.4 (3) Å^3^	0.2 × 0.2 × 0.2 mm
*Z* = 4	

### Data collection

**Table:**

Bruker SMART APEXII area-detector diffractometer	2564 reflections with *I* > 2σ(*I*)
Radiation source: fine-focus sealed tube	*R*_int_ = 0.035
graphite	θ_max_ = 28.3°, θ_min_ = 2.4°
ω and φ scans	*h* = −13→13
6913 measured reflections	*k* = −21→21
3051 independent reflections	*l* = −12→12

### Refinement

**Table:**

Refinement on *F*^2^	Secondary atom site location: difference Fourier map
Least-squares matrix: full	Hydrogen site location: inferred from neighbouring sites
*R*[*F*^2^ > 2σ(*F*^2^)] = 0.033	H-atom parameters constrained
*wR*(*F*^2^) = 0.081	*w* = 1/[σ^2^(*F*_o_^2^) + (0.0327*P*)^2^] where *P* = (*F*_o_^2^ + 2*F*_c_^2^)/3
*S* = 0.98	(Δ/σ)_max_ = 0.001
3051 reflections	Δρ_max_ = 0.50 e Å^−^^3^
184 parameters	Δρ_min_ = −0.37 e Å^−^^3^
2 restraints	Absolute structure: Flack (1983), 1147 Friedel pairs
Primary atom site location: structure-invariant direct methods	Flack parameter: 0.009 (9)

### Special details

**Table:**

Geometry. All esds (except the esd in the dihedral angle between two l.s. planes) are estimated using the full covariance matrix. The cell esds are taken into account individually in the estimation of esds in distances, angles and torsion angles; correlations between esds in cell parameters are only used when they are defined by crystal symmetry. An approximate (isotropic) treatment of cell esds is used for estimating esds involving l.s. planes.
Refinement. Refinement of F^2^ against ALL reflections. The weighted R-factor wR and goodness of fit S are based on F^2^, conventional R-factors R are based on F, with F set to zero for negative F^2^. The threshold expression of F^2^ > 2sigma(F^2^) is used only for calculating R-factors(gt) etc. and is not relevant to the choice of reflections for refinement. R-factors based on F^2^ are statistically about twice as large as those based on F, and R- factors based on ALL data will be even larger.

### Fractional atomic coordinates and isotropic or equivalent isotropic displacement parameters (Å^2^)

**Table:**

	*x*	*y*	*z*	*U*_iso_*/*U*_eq_	
C1	0.5566 (3)	0.96379 (15)	0.4642 (4)	0.0358 (6)	
C2	0.6217 (3)	1.03392 (15)	0.5239 (3)	0.0349 (6)	
C3	0.5803 (3)	1.11292 (14)	0.4782 (4)	0.0391 (7)	
H3	0.6233	1.1589	0.5178	0.047*	
C4	0.4795 (3)	1.12589 (15)	0.3781 (4)	0.0361 (6)	
C5	0.4150 (3)	1.05601 (17)	0.3224 (4)	0.0379 (7)	
H5	0.3454	1.0623	0.2554	0.045*	
C6	0.4536 (3)	0.97694 (16)	0.3657 (4)	0.0388 (7)	
C7	0.7280 (3)	1.02509 (19)	0.6298 (4)	0.0421 (8)	
H7	0.7676	1.0725	0.6684	0.051*	
C8	0.8724 (4)	0.94730 (17)	0.7759 (5)	0.0423 (7)	
C9	0.9091 (3)	0.8682 (2)	0.8210 (5)	0.0531 (9)	
H9	0.8653	0.8219	0.7837	0.064*	
C10	1.0103 (4)	0.8595 (3)	0.9207 (5)	0.0653 (11)	
H10	1.0367	0.8069	0.9510	0.078*	
C11	1.0721 (4)	0.9279 (3)	0.9754 (5)	0.0698 (11)	
H11	1.1401	0.9235	1.0447	0.084*	
C12	1.0307 (4)	1.0042 (3)	0.9248 (5)	0.0695 (13)	
H12	1.0734	1.0511	0.9615	0.083*	
C13	0.4388 (3)	1.2146 (2)	0.3348 (4)	0.0436 (8)	
C14	0.3815 (4)	1.2584 (2)	0.4631 (5)	0.0662 (10)	
H14A	0.3569	1.3140	0.4375	0.099*	
H14B	0.4475	1.2596	0.5394	0.099*	
H14C	0.3041	1.2289	0.4929	0.099*	
C15	0.5606 (3)	1.26226 (17)	0.2862 (5)	0.0564 (9)	
H15A	0.5982	1.2340	0.2074	0.085*	
H15B	0.6259	1.2656	0.3629	0.085*	
H15C	0.5342	1.3171	0.2576	0.085*	
C16	0.3336 (4)	1.2144 (3)	0.2134 (6)	0.0654 (12)	
H16A	0.3703	1.1896	0.1309	0.098*	
H16B	0.3071	1.2704	0.1921	0.098*	
H16C	0.2573	1.1832	0.2410	0.098*	
Br1	0.36263 (5)	0.884372 (16)	0.28725 (6)	0.06324 (14)	
N1	0.7694 (2)	0.95411 (16)	0.6724 (3)	0.0412 (6)	
N2	0.9331 (3)	1.01500 (19)	0.8263 (4)	0.0573 (8)	
O1	0.5940 (2)	0.88708 (9)	0.5015 (3)	0.0519 (7)	
H1	0.5462	0.8533	0.4592	0.078*	

### Atomic displacement parameters (Å^2^)

**Table:**

	*U*^11^	*U*^22^	*U*^33^	*U*^12^	*U*^13^	*U*^23^
C1	0.0336 (12)	0.0318 (11)	0.0417 (18)	0.0008 (10)	−0.0011 (12)	0.0018 (12)
C2	0.0341 (12)	0.0318 (11)	0.0383 (18)	0.0021 (10)	−0.0067 (12)	0.0026 (12)
C3	0.0421 (14)	0.0292 (12)	0.0452 (19)	0.0002 (10)	−0.0085 (14)	−0.0002 (12)
C4	0.0337 (12)	0.0357 (13)	0.0387 (18)	0.0014 (10)	−0.0023 (13)	0.0025 (13)
C5	0.0330 (13)	0.0400 (14)	0.0401 (18)	−0.0020 (10)	−0.0077 (12)	0.0009 (13)
C6	0.0383 (14)	0.0352 (12)	0.0428 (19)	−0.0049 (11)	0.0003 (13)	−0.0021 (13)
C7	0.0447 (16)	0.0343 (13)	0.047 (2)	0.0019 (11)	−0.0068 (15)	−0.0023 (14)
C8	0.0405 (15)	0.0473 (13)	0.0388 (19)	0.0049 (15)	−0.0022 (13)	0.0061 (18)
C9	0.0532 (19)	0.0542 (18)	0.052 (2)	0.0096 (13)	−0.0039 (16)	0.0086 (16)
C10	0.060 (2)	0.076 (2)	0.059 (3)	0.0227 (19)	−0.003 (2)	0.026 (2)
C11	0.0523 (19)	0.101 (3)	0.055 (2)	0.011 (2)	−0.0214 (17)	0.015 (2)
C12	0.060 (2)	0.081 (3)	0.066 (3)	−0.0077 (19)	−0.024 (2)	0.010 (2)
C13	0.0484 (17)	0.0345 (15)	0.047 (2)	0.0037 (13)	−0.0061 (16)	0.0045 (14)
C14	0.076 (2)	0.0498 (18)	0.074 (3)	0.0211 (16)	0.013 (2)	0.0060 (18)
C15	0.067 (2)	0.0377 (14)	0.064 (3)	−0.0013 (13)	−0.0035 (18)	0.0117 (15)
C16	0.067 (3)	0.051 (2)	0.077 (3)	0.0112 (17)	−0.024 (2)	0.011 (2)
Br1	0.0703 (2)	0.04400 (16)	0.0735 (3)	−0.01579 (16)	−0.02473 (16)	−0.0033 (2)
N1	0.0381 (12)	0.0418 (13)	0.0431 (17)	0.0045 (10)	−0.0089 (11)	0.0036 (11)
N2	0.0525 (15)	0.0573 (16)	0.060 (2)	−0.0037 (12)	−0.0218 (14)	0.0070 (15)
O1	0.0612 (14)	0.0255 (9)	0.0679 (19)	−0.0005 (8)	−0.0146 (13)	0.0030 (9)

### Geometric parameters (Å, °)

**Table:**

C1—O1	1.337 (3)	C10—C11	1.359 (7)
C1—C6	1.380 (4)	C10—H10	0.9300
C1—C2	1.413 (3)	C11—C12	1.378 (6)
C2—C3	1.404 (3)	C11—H11	0.9300
C2—C7	1.441 (5)	C12—N2	1.336 (5)
C3—C4	1.373 (5)	C12—H12	0.9300
C3—H3	0.9300	C13—C16	1.529 (5)
C4—C5	1.393 (4)	C13—C15	1.527 (5)
C4—C13	1.540 (4)	C13—C14	1.531 (6)
C5—C6	1.390 (4)	C14—H14A	0.9600
C5—H5	0.9300	C14—H14B	0.9600
C6—Br1	1.887 (3)	C14—H14C	0.9600
C7—N1	1.278 (4)	C15—H15A	0.9600
C7—H7	0.9300	C15—H15B	0.9600
C8—N2	1.330 (5)	C15—H15C	0.9600
C8—C9	1.390 (4)	C16—H16A	0.9600
C8—N1	1.399 (5)	C16—H16B	0.9600
C9—C10	1.366 (6)	C16—H16C	0.9600
C9—H9	0.9300	O1—H1	0.8200
			
O1—C1—C6	121.0 (3)	C12—C11—H11	121.1
O1—C1—C2	121.0 (3)	N2—C12—C11	124.2 (4)
C6—C1—C2	117.9 (2)	N2—C12—H12	117.9
C3—C2—C1	118.5 (3)	C11—C12—H12	117.9
C3—C2—C7	120.4 (3)	C16—C13—C15	108.3 (3)
C1—C2—C7	121.1 (2)	C16—C13—C14	108.9 (4)
C4—C3—C2	123.5 (2)	C15—C13—C14	109.4 (3)
C4—C3—H3	118.3	C16—C13—C4	111.5 (3)
C2—C3—H3	118.3	C15—C13—C4	110.0 (3)
C3—C4—C5	117.1 (2)	C14—C13—C4	108.8 (3)
C3—C4—C13	120.4 (3)	C13—C14—H14A	109.5
C5—C4—C13	122.5 (3)	C13—C14—H14B	109.5
C6—C5—C4	120.8 (3)	H14A—C14—H14B	109.5
C6—C5—H5	119.6	C13—C14—H14C	109.5
C4—C5—H5	119.6	H14A—C14—H14C	109.5
C1—C6—C5	122.2 (3)	H14B—C14—H14C	109.5
C1—C6—Br1	118.8 (2)	C13—C15—H15A	109.5
C5—C6—Br1	119.1 (2)	C13—C15—H15B	109.5
N1—C7—C2	122.0 (3)	H15A—C15—H15B	109.5
N1—C7—H7	119.0	C13—C15—H15C	109.5
C2—C7—H7	119.0	H15A—C15—H15C	109.5
N2—C8—C9	122.1 (4)	H15B—C15—H15C	109.5
N2—C8—N1	120.1 (3)	C13—C16—H16A	109.5
C9—C8—N1	117.8 (3)	C13—C16—H16B	109.5
C10—C9—C8	119.2 (4)	H16A—C16—H16B	109.5
C10—C9—H9	120.4	C13—C16—H16C	109.5
C8—C9—H9	120.4	H16A—C16—H16C	109.5
C9—C10—C11	119.6 (4)	H16B—C16—H16C	109.5
C9—C10—H10	120.2	C7—N1—C8	120.9 (3)
C11—C10—H10	120.2	C8—N2—C12	117.1 (4)
C10—C11—C12	117.8 (4)	C1—O1—H1	109.5
C10—C11—H11	121.1		
			
O1—C1—C2—C3	178.5 (3)	N2—C8—C9—C10	0.3 (6)
C6—C1—C2—C3	−1.2 (4)	N1—C8—C9—C10	179.3 (3)
O1—C1—C2—C7	−1.8 (4)	C8—C9—C10—C11	0.9 (6)
C6—C1—C2—C7	178.5 (3)	C9—C10—C11—C12	−1.2 (6)
C1—C2—C3—C4	−0.1 (5)	C10—C11—C12—N2	0.5 (7)
C7—C2—C3—C4	−179.8 (3)	C3—C4—C13—C16	175.1 (4)
C2—C3—C4—C5	1.1 (5)	C5—C4—C13—C16	−6.8 (5)
C2—C3—C4—C13	179.4 (3)	C3—C4—C13—C15	55.0 (5)
C3—C4—C5—C6	−1.0 (5)	C5—C4—C13—C15	−126.9 (3)
C13—C4—C5—C6	−179.1 (3)	C3—C4—C13—C14	−64.8 (4)
O1—C1—C6—C5	−178.3 (3)	C5—C4—C13—C14	113.3 (4)
C2—C1—C6—C5	1.4 (5)	C2—C7—N1—C8	−179.8 (3)
O1—C1—C6—Br1	1.6 (4)	N2—C8—N1—C7	−3.3 (5)
C2—C1—C6—Br1	−178.7 (2)	C9—C8—N1—C7	177.7 (3)
C4—C5—C6—C1	−0.3 (5)	C9—C8—N2—C12	−1.0 (6)
C4—C5—C6—Br1	179.8 (2)	N1—C8—N2—C12	−180.0 (3)
C3—C2—C7—N1	−179.1 (3)	C11—C12—N2—C8	0.6 (6)
C1—C2—C7—N1	1.3 (5)		

### Hydrogen-bond geometry (Å, °)

**Table:**

*D*—H···*A*	*D*—H	H···*A*	*D*···*A*	*D*—H···*A*
O1—H1···Br1	0.82	2.46	3.021 (3)	127
C7—H7···N2	0.93	2.38	2.723 (5)	102
